# Cystic Lymphangioma of the Greater Omentum: A Case of Partial Spontaneous Regression and Review of the Literature

**DOI:** 10.1155/2020/8932017

**Published:** 2020-01-28

**Authors:** Haraesh Maranna, Lovenish Bains, Pawan Lal, Rahul Bhatia, Mohd Yasir Beg, Pritesh Kumar, Varuna Mallya

**Affiliations:** ^1^Department of Surgery, Maulana Azad Medical College, New Delhi, India; ^2^Department of Pathology, Maulana Azad Medical College, New Delhi, India

## Abstract

*Introduction*. Omental cysts are a part of cystic lymphangiomas and are benign proliferations of ectopic lymphatics without a communication with the normal lymphatic system. They commonly involve the neck in the pediatric population and are uncommon at other sites and occur rarely in adults. *Case Presentation*. A 42-year-old female with complaints of vague lower abdominal pain for 8 months presented with a soft, nontender swelling of size 22 × 18 cm in the hypogastrium and umbilical region. Computerized tomography (CT) of the abdomen showed a peripherally enhancing hypodense cystic lesion of size 19 × 14 × 12 cm perhaps arising from the mesentery. The cyst had spontaneously reduced in size by about 70% over the next 4 months. During surgery, the cyst of size 10 × 9 × 8 cm was present in the greater omentum. Excision was done, and histopathology was suggestive of cystic lymphangioma. *Discussion*. Cystic lymphangiomas have an incidence of 1/20000 at infancy and 1/100000 to 1/250000 of hospital admissions in adults, and the female-to-male ratio is 2 : 1. In adults, they are found in the age group between 40 and 70 years. Spontaneous regression of omental cysts is very rare and presumably from increased pressure in cysts overcoming incomplete obstructions or by establishment of alternative routes of drainage.

**Conclusion:**

As the disease is essentially benign and if there are no significant pressure symptoms, the cysts of short duration can be watched further for regression. Long-standing, symptomatic cysts, nonregression, and diagnostic uncertainty will warrant surgery to confirm the diagnosis and relieve the symptoms.

## 1. Introduction

Omental cysts are a part of cystic lymphangiomas and are benign proliferations of ectopic lymphatics that do not have communication with the normal lymphatic system [1]. They occur usually in the pediatric age group and are rare in adults. We report our experience of a huge omental cyst in a 42-year-old female who underwent more than 70% spontaneous regression.

## 2. Case Presentation

A 42-year-old female presented with a painless swelling in the lower abdomen for duration of 8 months, gradually increasing in size. There were no bladder, bowel, or any gynecological complaints. There was no history of trauma or previous abdominal surgery. She was diabetic, hypertensive, and hypothyroid on regular medications. The menstrual cycles were regular, and she had two full-term normal vaginal deliveries. Her family history and personal history were unremarkable. On examination, vital parameters were normal. On examination, the abdomen was distended and the umbilicus everted and deviated upwards. A soft, nontender, smooth swelling of 22 × 18 cm along the horizontal and longitudinal axis, respectively, was palpable in the hypogastrium and umbilical regions showing some mobility. Per-vaginal and per-rectal examination was normal.

**Figure 1:**
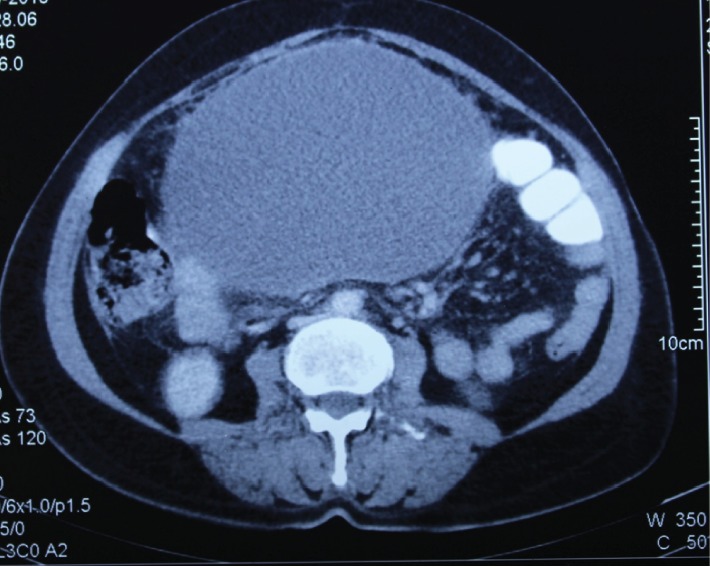
CT transverse plane of the abdomen showing a huge cystic lesion of 19 × 14 × 12 cm.

**Figure 2:**
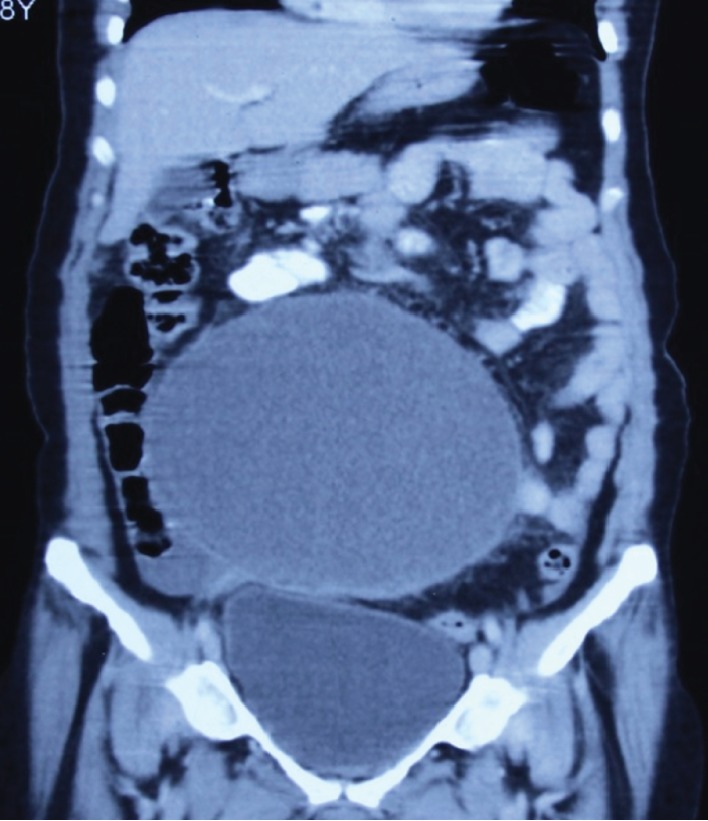
CT coronal plane showing the huge cyst displacing the small-bowel loops superiorly and laterally.

An ultrasonogram (USG) revealed an anechoic cystic lesion of size 17 × 16 × 12 cm with no obvious septations and probably arising from the right adnexa, with the normal left adnexa and uterus. A CT scan of the abdomen showed a peripherally enhancing hypodense cystic lesion of size 19 × 14 × 12 cm arising from the mesentery displacing the small-bowel loops superiorly and laterally (Figures 1 and 2). There were no solid components or calcifications present. The uterus and adnexal organs were normal. Based on examination and investigations, a differential diagnosis of an omental cyst, mesenteric cyst, hydatid cyst, or tuboovarian mass was made.

**Figure 3:**
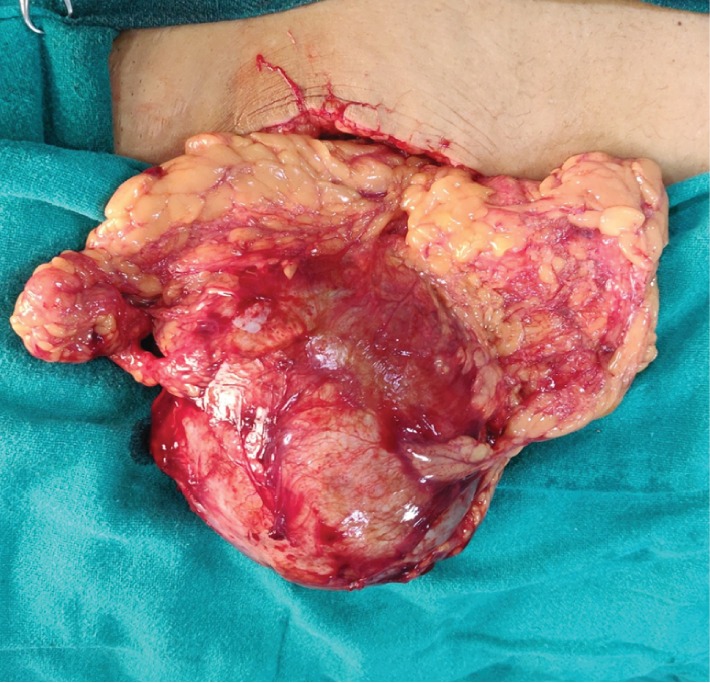
Intraoperative image showing the huge omental cyst wrapped in the greater omentum.

**Figure 4:**
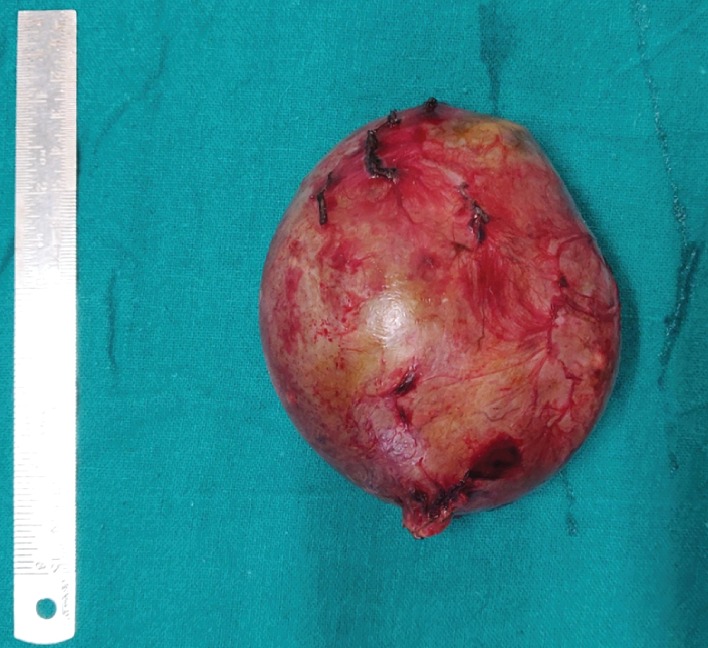
Excised omental cyst of size 8 × 8 cm.

**Figure 5:**
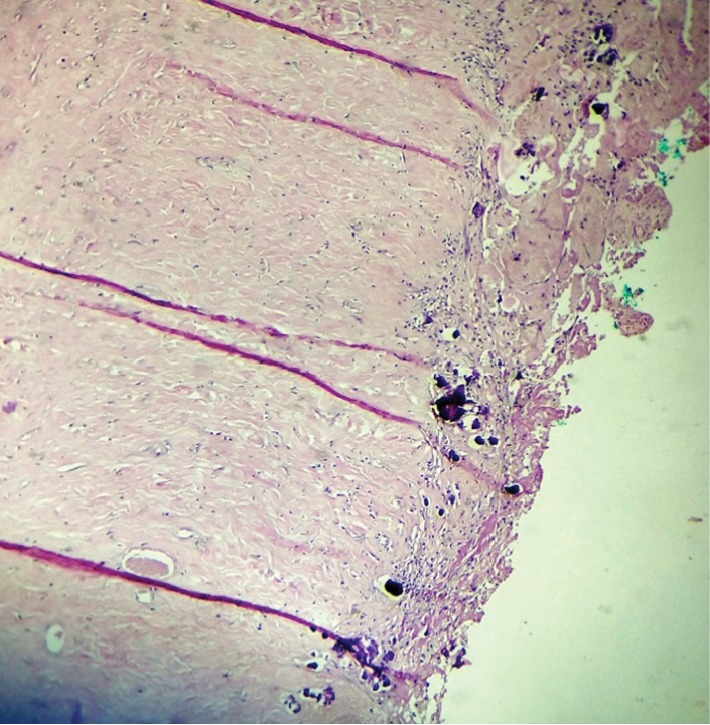
Photomicrograph showing foci of calcification (hematoxylin and eosin, ×400).

**Figure 6:**
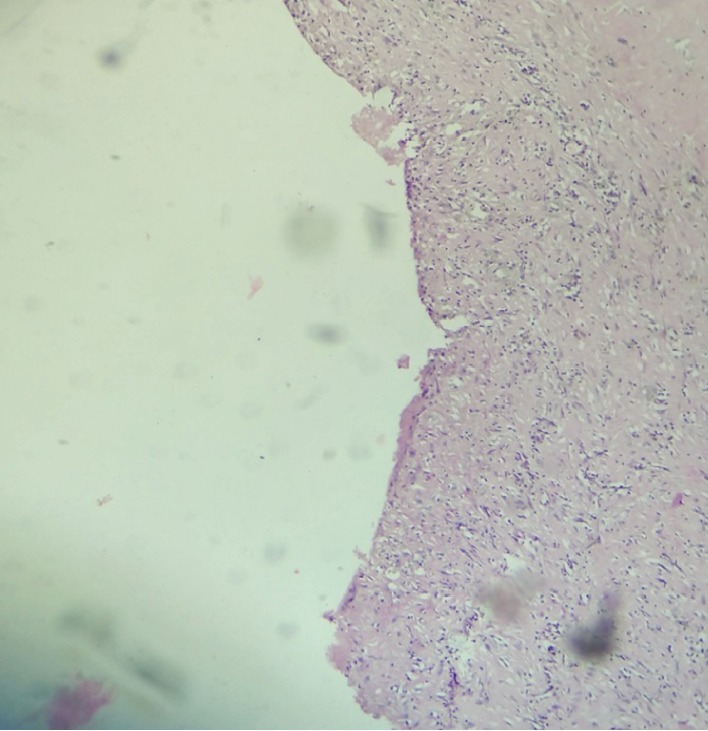
Photomicrograph showing the wall of the cyst lined by flattened cells (hematoxylin and eosin, ×400).

Her laboratory investigations revealed hemoglobin of 10.2 g% and normal liver and renal function tests. CA 125 (cancer antigen) levels were 12 units/ml (reference range 0-35 units/ml), and carcinoembryonic antigen (CEA) levels were 2.2 ng/ml (reference range 0-3.8 ng/ml). Hydatid serology was negative. The patient did not report for the next 4 months owing to some tragedy in the family. There was no trauma or history suggestive of possible rupture during that period. This time the swelling was 8 cm in size, well defined, and mobile in the umbilical region. Repeat of USG showed a decrease in the size of the cyst to 10 × 9 × 9 cm, undergoing spontaneous regression of more than 70%. Since there was a reduction in cyst size, the patient was observed for a further period of three months, and as no further reduction was noted, the patient was planned for surgery. The patient underwent laparotomy through a 10 cm lower midline vertical incision. During surgery, a well-defined cyst of size 10 × 9 × 8 cm was present wrapped in the greater omentum predominantly right side and approximately 15 cm away from the transverse colon (Figures 3 and 4). The greater omental cyst was not adhered to the bowel, adnexa, or any other surrounding structures. No other lesions or cysts were noted in the omentum and mesentery. There were no grossly visible dilated channels or possibly fibrosed and obliterated lymphatic channels as there was no further reduction in size of the cyst despite observation for 3 months. Excision of the omental cyst along with the cuff of the omentum was done using a combination of ligatures and LigaSure (Valleylab, Boulder, CO, USA). No significant bleeding was observed and no drains were placed. A postoperative period was uneventful, and the patient was discharged on day 4. Histopathological examination revealed a wall of a cyst lined by flattened cells and areas showing foci of calcification suggestive of cystic lymphangioma (Figures 5 and 6). The patient underwent an USG at 6 months which was normal and is healthy for more than 1 year of follow-up.

## 3. Discussion

Omental and mesenteric cysts are uncommon benign tumors usually occurring in children with an incidence of 1/20000 at infancy and 1/100000 to 1/250000 of hospital admissions in adults [1]. Sixty-five percent of cases are present at birth, and the female-to-male ratio is 2 : 1 [2]. In adults, they are rare and found in the age group between 40 and 70 years [3]. In 1507, the Italian anatomist Benevieni first reported a mesenteric cyst following an autopsy on an 8-year-old child and the first description of a chylous cyst in an autopsy was recorded by Rokitansky in 1842 [4]. Gairdner published the first report of an omental cyst in 1852, and in 1880, Tillaux performed the first successful operation on a cystic mesenteric tumor [4]. Cystic lymphangiomas can occur anywhere in the body, most commonly occurring in the neck (75%), axilla (20%), and other organ sites such as the esophagus, liver, spleen, and mediastinum and other abdominal organs (4%) [5]. In 1% of cases, they are found in the mesentery out of which 85% occur in the mesentery of the small bowel, 10% in the mesocolon, and 5% in the retroperitoneum [6]. They are usually asymptomatic and found incidentally on USG or CT scan or during laparotomy done for other conditions [7]. The common clinical presentations of larger cysts include vague abdominal pain and abdominal distension sometimes mimicking ascites [3]. Larger cysts can present with acute symptoms of pain and peritonitis due to rupture, infection, hemorrhage into the cyst, torsion, volvulus, or intestinal obstruction secondary to the cyst [7, 8]. The cysts may be single or multiple. The cysts may be filled with serous, chylous, infected, or hemorrhagic content. In mesenteric and omental cysts, 10% of patients present clinically as having acute abdomen and these are almost exclusively found in childhood [3, 4, 9].

The mechanisms of formation of cystic lymphangiomas include (i) failure of embryonic channels to join the venous system, (ii) trauma, (iii) neoplasm, (iv) failure of the leaves of mesentery to fuse, (v) and degeneration of lymph nodes [3, 4, 7]. Traumatic or infectious cysts usually have a fibrous wall, cholesterol granuloma, foamy cells, and absence of epithelium lining and may histologically resemble pancreatic pseudocysts. Malignant cysts are very rare with a reported incidence of less than 3% [10]. The largest omental cyst reported was of size 33 × 30 × 25 cm and weighed 16 kg [11].

In our case, we observed the reduction in size of the cyst of the greater omentum by more than 70% over the period of 4 months. There have been very few cases reported with spontaneous regression of lymphangiomas [12, 13]. Lee described multiple lymphangiomas of the colon in a 54-year-old male diagnosed by colonoscopy and mucosal resection and observed spontaneous resolution 12 months after the initial presentation [12]. Joo et al. diagnosed a case of retroperitoneal cystic lymphangioma of 11 cm size in an 18-year-old postpartum lady. The cyst gradually reduced in size and was no longer visible 2 years after the initial presentation [13]. In our case, we proceeded with excision of the cyst after observation for 3 months due the diagnostic uncertainty associated with it.

The hypothesis developed for the spontaneous regression of cystic hygromas of the neck and spleen is that increased pressure in the lymphatic system may overcome incomplete obstructions. Spontaneous resolution presumably results from the establishment of alternative routes of lymphatic drainage. The leakage induced by increased pressure of the cyst may stimulate dendritic cells or macrophages in the stroma. In a prolonged state of lymphatic stagnation, CD68-positive cells, which can be dendritic cells or macrophages, might correspond to the cells equipped with phagosomes and lysosomes. These stimulated cells would evidence phagocytic activity on the lining cells and persist until the lymphangioma is resolved [12]. Hence, a more conservative approach to omental cysts can be attempted in the absence of complications and can be watched for a period of 6 months to 1 year.

Ultrasound of the abdomen is the initial modality of choice in suspected cysts which may demonstrate a large, unilocular, or septated nonspecific cystic lesion with regular contours. Lymphangiomas are anechoic cysts with posterior acoustic enhancement [14]. They must be differentiated from ascites by the absence of fluid in the dependent regions and by the absence of bowel loop separation [15]. Plain radiographs are not useful in the diagnosis of omental cysts. They may show a noncalcified soft tissue mass, small-bowel obstruction, and displacement of bowel loops in larger cysts. Contrast-enhanced computerized tomography (CECT) of the abdomen provides more information regarding location, extent, and nature of the cyst whereas a CT scan with oral contrast helps show the relationship of the cyst with the intestine and surrounding structures [16].

Surgical excision is the gold standard treatment [17]. Malignant degeneration to low-grade sarcoma has been reported but is rare; however, complete surgical excision is the treatment of choice [8, 18]. Although laparoscopic excision of the cysts has been successfully reported, an open method of excision is still the preferred approach [19–21]. Percutaneous aspiration has been tried, but infection and recurrence rates have been high [22]. Other treatment options include external marsupialization and internal drainage, both of which are associated with a high rate of morbidity and risk of recurrence [7].

## 4. Conclusion

Omental cysts are rare intra-abdominal benign tumors occurring commonly in childhood but rarer in adults. Small cysts are asymptomatic where larger ones present with a vague abdominal lump and distension. Since the disease is essentially benign and if there are no significant pressure symptoms, the cysts can be watched for a period of 6-12 months. Symptomatic cysts, nonregression, and diagnostic uncertainty will warrant surgery to confirm the diagnosis and relieve the symptoms.

## Abbreviations

CT: Computerized tomography
USG: Ultrasonogram
CA: Cancer antigen
CEA: Carcinoembryonic antigen
CECT: Contrast-enhanced computerized tomography.
